# Gut-derived immune cells and the gut-lung axis in ARDS

**DOI:** 10.1186/s13054-024-05006-x

**Published:** 2024-07-04

**Authors:** Mairi Ziaka, Aristomenis Exadaktylos

**Affiliations:** 1 Clinic of Geriatric Medicine, Center of Geriatric Medicine and Rehabilitation, Kantonsspital Baselland, Bruderholz, Switzerland; 2 Department of Emergency Medicine, Inselspital, University Hospital, University of Bern, Bern, Switzerland

## Abstract

The gut serves as a vital immunological organ orchestrating immune responses and influencing distant mucosal sites, notably the respiratory mucosa. It is increasingly recognized as a central driver of critical illnesses, with intestinal hyperpermeability facilitating bacterial translocation, systemic inflammation, and organ damage. The “gut-lung” axis emerges as a pivotal pathway, where gut-derived injurious factors trigger acute lung injury (ALI) through the systemic circulation. Direct and indirect effects of gut microbiota significantly impact immune responses. Dysbiosis, particularly intestinal dysbiosis, termed as an imbalance of microbial species and a reduction in microbial diversity within certain bodily microbiomes, influences adaptive immune responses, including differentiating T regulatory cells (Tregs) and T helper 17 (Th17) cells, which are critical in various lung inflammatory conditions. Additionally, gut and bone marrow immune cells impact pulmonary immune activity, underscoring the complex gut-lung interplay. Moreover, lung microbiota alterations are implicated in diverse gut pathologies, affecting local and systemic immune landscapes. Notably, lung dysbiosis can reciprocally influence gut microbiota composition, indicating bidirectional gut-lung communication. In this review, we investigate the pathophysiology of ALI/acute respiratory distress syndrome (ARDS), elucidating the role of immune cells in the gut-lung axis based on recent experimental and clinical research. This exploration aims to enhance understanding of ALI/ARDS pathogenesis and to underscore the significance of gut-lung interactions in respiratory diseases.

## Introduction

The intestine represents an expansive mucosal surface, positioning itself as the largest immunological organ, with immune cells distributed throughout both the lamina propria and epithelium of the intestinal mucosa. Playing the role of a major immune organ, it orchestrates immune responses upon confronting antigens and extends its influence to distant mucosal sites, particularly the respiratory mucosa [1].

Moreover, over the last decades, it has been postulated that the gastrointestinal (GI) tract serves as the central driver of critical illness, characterized by intestinal hyperpermeability, which facilitates bacterial translocation through the portal circulation and mesenteric lymphatics, culminating in subsequent systemic infection and damage to distant organs acting as the principal conduit for gut-derived injurious factors which potentially may trigger ARDS [2–4]. This substantiation lies in the lung’s initial encounter with the mesenteric lymph, where it is exposed to a high concentration of lymph contents before dilution by the systemic blood volume. Due to the fact that intestinal lymph enters the systemic circulation via the thoracic duct, which empties into the subclavian vein and subsequently reaches the right heart before being pumped into the pulmonary circulation, the pulmonary vascular bed is the first site to encounter mesenteric lymph [3]. Moreover, damage to the intestinal mucosa can result in the production of harmful substances such as endotoxins, microbial metabolites, and hormones or inflammatory mediators, disrupting the integrity of the gut barrier. Once these substances are absorbed into the mesenteric lymph duct, they enter the bloodstream and activate endothelial [5] and immune cells, ultimately leading to ALI [5, 6].

Various pathways of communication have been recognized within the gut-lung axis. These pathways include direct effects of gut microbiota, illustrated by the enhancement of the host’s immune response through the presence of peptidoglycan and lipopolysaccharide (LPS) [7]. Referring to the complex ecosystems of microorganisms inhabiting the intestinal tract, the term “gut microbiota” encompasses over 1000 types of microorganisms, spanning at least 4000 distinct species [8–11]. Additionally, indirect effects of gut microbiota are observed, such as unmetabolized short-chain fatty acids (SCFAs) influencing the development of immune cells upon entering the peripheral bloodstream [7]. Shaping adaptive immune responses, particularly the development and differentiation of CD4+ and CD8+ T cells, is another role of the gut microbiota. Dysbiosis triggers and activates Tregs, fosters the proliferation and differentiation of Tregs and Th17 cells, resulting in the production of interleukin (IL)-17 by intestinal Th17 cells, and prompts B cells to produce and release secretory immunoglobulin A (IgA) [12, 13]. Recent research indicates that immune cells, including Th17 and Treg cells, which primarily originate from progenitor cells in the bone marrow and among others from the gut, play a central role in various lung inflammatory pathologies such as chronic obstructive pulmonary disease (COPD), ARDS, sarcoidosis, asthma, and pulmonary infectious diseases [14–16]. Additionally, immune cells originating from the bone marrow trigger an immune response in the lungs, while intestinal immune cells migrate directly from the intestinal tract to the lungs via the bloodstream, impacting pulmonary immune activity [7].

Moreover, contrary to the long-standing notion of lung sterility, which has hindered systematic exploration of the lung microbiome and research progress, it is now firmly established that even healthy, asymptomatic individuals undergo colonization of alveoli by microbes [17]. Our knowledge about lung microbiota has been enhanced by improvements in both culture‐dependent and ‐independent techniques, aiding understanding not only of its presence in healthy lung tissue but also its significant impact on immune responses in both physiological and pathological states [18]. Although lung microbiota compositions in healthy individuals remain relatively stable, various clinical pathologies, including idiopathic pulmonary fibrosis (IPF), asthma, COPD, cystic fibrosis, cancer, and ALI/ARDS are associated with numerous patterns of lung dysbiosis [15, 19–25]. Furthermore, it is now well established that alterations in lung microbiota significantly affect both the local pulmonary and systemic landscape of immune cells [26], setting the stage for a feedback loop where local immune cells and microbiota engage in bidirectional communication [27]. Interestingly, alterations in lung microbiota may also influence the composition of gut microbiota [15, 26] in the context of dynamic crosstalk between gut and lung [27].

In the present review, we aim to further clarify the pathophysiology of ALI/ARDS by describing the various effects of immune cells within the gut-lung axis in patients with ARDS based on recent experimental and clinical data. While our focus is on elucidating shared pathophysiological mechanisms that critically influence the gut-lung axis, it is important to note that the variable effects of injury models (direct vs. indirect) and insult types (infectious vs. non-infectious) on disease outcomes and underlying mechanisms should be acknowledged.

## The gut-lung *axis* during critical illness

Intensifying interest in biomedical research has emerged due to the recognition that gut microbiota functions as a vital superorganism and an autonomous organ, impacting various physiological processes in the host [28], including protection against enteric and systemic pathogens [29] by pleiotropic mechanisms as for example maintenance of intestinal epithelial integrity, nutritional competition and, immune system modulation [15, 30–33]. The term “gut microbiota” encompasses over 1000 types of microorganisms and at least 4000 distinct species, referring to the complex communities of commensal and pathogenic microorganisms residing in the intestinal tract [8–11] including bacteria, viruses, fungi, and parasites [34]. Despite normally engaging in a hormonally symbiotic relationship with the host under physiologic conditions, the mammalian gut microbiome undergoes a rapid decline in microbial density, membership composition, and comprehensive community structure and function within hours following a sudden physiological insult [32, 35] termed as “pathobiome” [36].

Emerging experimental and epidemiological evidence has underscored a critical interplay between the intestinal microbiota and the lungs, recognized as the “gut-lung axis” [37]. This concept reflects the anatomical independence of the gut and lungs yet emphasizes the existence of complex interactions in both health and disease. These interactions involve not only the host-microbe relationship but also the crosstalk among different microbial communities, which influence local and systemic immune responses and airway homeostasis [37, 38]. The gut-lung axis functions through both local regulatory mechanisms and distant effects, impacting the progression of respiratory diseases by modulating immune and inflammatory responses compromising the integrity of the intestinal barrier which is associated with bacterial translocation, lung injury, sustained inflammation, and pulmonary fibrosis [37, 39–42].

### Gut microbiome

Maintaining gut health heavily depends on the intestinal mucosal barrier, which balances the microbiota through antimicrobial peptides and the coordinated actions of the mucous layer and cell junctions [43]. During critical illnesses, the gut is notably vulnerable to injury, with approximately 50% of intensive care unit (ICU) patients experiencing enterocyte damage [44, 45]. Hypoperfusion and subsequent reperfusion of the intestinal wall, often induced by catecholamine administration to treat shock, sepsis, and robust non-infectious inflammation, can lead to reduced microvascular perfusion, severe mucosal inflammation, increased apoptosis, decreased proliferation of small bowel mucosal cells resulting to the compromise of the integrity of the intestinal mucus layer, decreased absorptive capacity, increased paracellular permeability and, alterations in systemic inflammation [46–52]. Moreover, intensive care settings commonly employ various clinical interventions, including enteral feeding, administration of proton-pump inhibitors, systemic catecholamines, and systemic antibiotics, all of which alter the environmental growth conditions for intestinal bacteria [53].

The mucus layer, an essential anatomical component of gut anatomy, acts as a physical barrier that hosts its own protective microbiota, separating the intestinal ecosystem from the host [53]. The resulting outcome of the aforementioned pathophysiologic alterations is an unstable and often decreased microbial diversity (as few as four species identified in some critically ill patients) [54, 55] with depletion of commensal microbes and proliferation of potentially pathogenic and inflammatory bacteria increasing susceptibility to hospital-acquired infections, sepsis, and multiple organ dysfunction syndrome (MODS) [56–58]. The upper GI tract, typically characterized by sparse microbial populations, becomes dominated by a limited number of species, such as *Escherichia coli* (*E. coli*), *Enterococcus spp*., and *Pseudomonas aeruginosa* (*Ps. aeruginosa*) [59, 60]. Moreover, during the first week in the ICU, critically ill patients showed a significantly different composition of intestinal microbiota compared to healthy subjects, with an increase in intestinal *Enterobacteriales* and *Enterobacteriaceae* associated with a 92% higher risk of 180-day adjusted mortality [61]. Freedberg and co-workers (2018) highlighted the predominance of pathogens such as *E. coli*, *Pseudomonas species*, *Klebsiella species*, and *Clostridium difficile* (*Cl. difficile*) in rectal swabs of 301 adult ICU patients. Additionally, an increased risk of death or all-cause infection was linked to the presence of *Enterococcus* as the predominant pathogen [62]. Furthermore, in a study of 81 patients with severe systemic inflammatory response syndrome (SIRS), the analysis of gut flora revealed that the complication of enteritis was primarily associated with a reduction in total obligate anaerobes and an increase in *Staphylococcus* and *Enterococcus* [63]. Finally, in an experimental study, fecal microbiota from patients with ARDS caused by community-acquired pneumonia (CAP) was transferred to antibiotics-treated recipient male mice, revealing that the intestinal flora of ARDS/CAP patients exhibited higher abundances of Gram-negative bacteria compared to normal controls. Additionally, mice that received fecal transplants from ARDS/CAP patients exhibited increased systemic LPS, systemic inflammation, and intestinal permeability. Interestingly, the altered gut microbiota from ARDS/CAP patients resulted in neuroinflammation and behavioral disturbances in mice [64].

### Lung microbiome

Under normal conditions, the alveolar space, unlike the GI tract, provides an environment that is generally hostile to most bacteria, resulting in minimal bacterial growth [53], mainly including *Prevotella*, *Veillonella*, *Streptococcus*, and *Fussobacterium species* [65–67]. However, critical illness leads to lung dysbiosis by altering local physiochemical and metabolic characteristics within the alveoli, such as pH levels, oxygen concentration, free radical occurrence, and nutrient availability [68–70]. Indeed, in pathological conditions such as ARDS, the previously empty alveolar airspaces are filled with protein-rich fluid, providing a favorable energy milieu for proliferating microorganisms [69]. Moreover, the development of anaerobic zones due to alveolar oedema or collapse, and consequent atelectasis in injured lungs, creates a more favorable environment for the proliferation of potential pathogens [68], exhibiting characteristics more similar to the gut environment than to healthy lung tissue [53]. Indeed, in patients with ARDS, Panzer and colleagues (2018) found an enrichment of the lung microbiome with gut-associated microbes, such as *Bacteroidetes* and *Enterobacteriaceae*. Additionally, the study revealed that in patients with severe blunt trauma undergoing mechanical ventilation, early alterations of the lung microbiome correlate with elevated markers of inflammation (IL-6, IL-8), making these patients susceptible to the development of ARDS [71]. These findings are in line with the findings of Dickson et al., who investigated the bacterial composition of lung samples obtained via bronchoalveolar lavage (BAL) from both ARDS and non-ARDS patients. *Streptococcaceae*, *Veillonellaceae*, *Prevotellaceae*, *Verrucomicrobiaceae*, and *Flavobacteriaceae* were identified as the dominant species in patients without ARDS. In contrast, BAL specimens from ARDS patients revealed the presence of *Pasteurellaceae* and *Enterobacteriaceae* [25].

As previously noted, the development of ARDS has been strongly correlated with gut-associated bacteria in the lungs. Yet, limited data exists on the consistency of microbiota changes in both the gut and lungs. Conducting 16S ribosomal RNA (rRNA) sequencing analysis on 26 patients with acute pancreatitis-induced ARDS, a study found that alterations in gut microbiota mirror those observed in lung microbiota from earlier studies, suggesting that the translocation of gut microbiota may result in changes in lung microbiota [72]. The transfer of gut microbiota to the lungs can occur through several mechanisms. Impaired intestinal permeability, as suggested by recent studies, allows gut microbes like *Bacteroidetes* and *Enterobacteriaceae* to translocate into the lungs of ARDS patients [71]. However, it is crucial to understand that gut-derived critical illness extends beyond bacterial translocation, as evidenced by experimental studies of trauma or haemorrhagic shock where post-shock mesenteric lymph shows an absence of bacterial 16 s rRNA genetic material but an increase in damage-associated molecular pattern material (DAMPs), indicating the transport of pro-inflammatory mediators to distant organs, especially the lungs [73]. The invasion of native gut bacteria through the intestinal mucosa into normally sterile tissues, potentially causing disease, is referred to as "bacterial translocation," a process that also includes the infiltration and movement of inflammatory molecules produced at the intestinal wall or toxic products from the intestine, potentially resulting in systemic damage [74]. Indeed, local activation of the mucosal immune system (MIS) is triggered by bacterial translocation, leading to the production of inflammatory mediators known as DAMPs, which travel through the mesenteric lymphatics to the lungs and systemic circulation. Innate immune cells recognize these molecules, which further stimulate pro-inflammatory pathways that hasten organ damage and the development of MODS [30].

## Immune cells within the gut-lung *axis*: impact of gut-derived immune cells in the pathophysiology of ALI/ARDS

As mentioned above, the gut microbiota contributes significantly to maintaining immune homeostasis by modulating the balance between pro-inflammatory Th17 and anti-inflammatory Treg cells in the GI tract [75]. At the same time, an imbalanced microbiome has been linked to an elevation in CD4+ -IL-17 cells within the gut [76]. These immune cell-mediated regulatory properties of the microbiota are not limited to the local intestinal level but extend to peripheral organs and systems such as the brain [77–79], lungs [25, 80, 81], and liver [82] through T cell migration [83, 84] underscoring a direct link within the gut-lung axis.

### Th17/Tregs

**Fig. 1:**
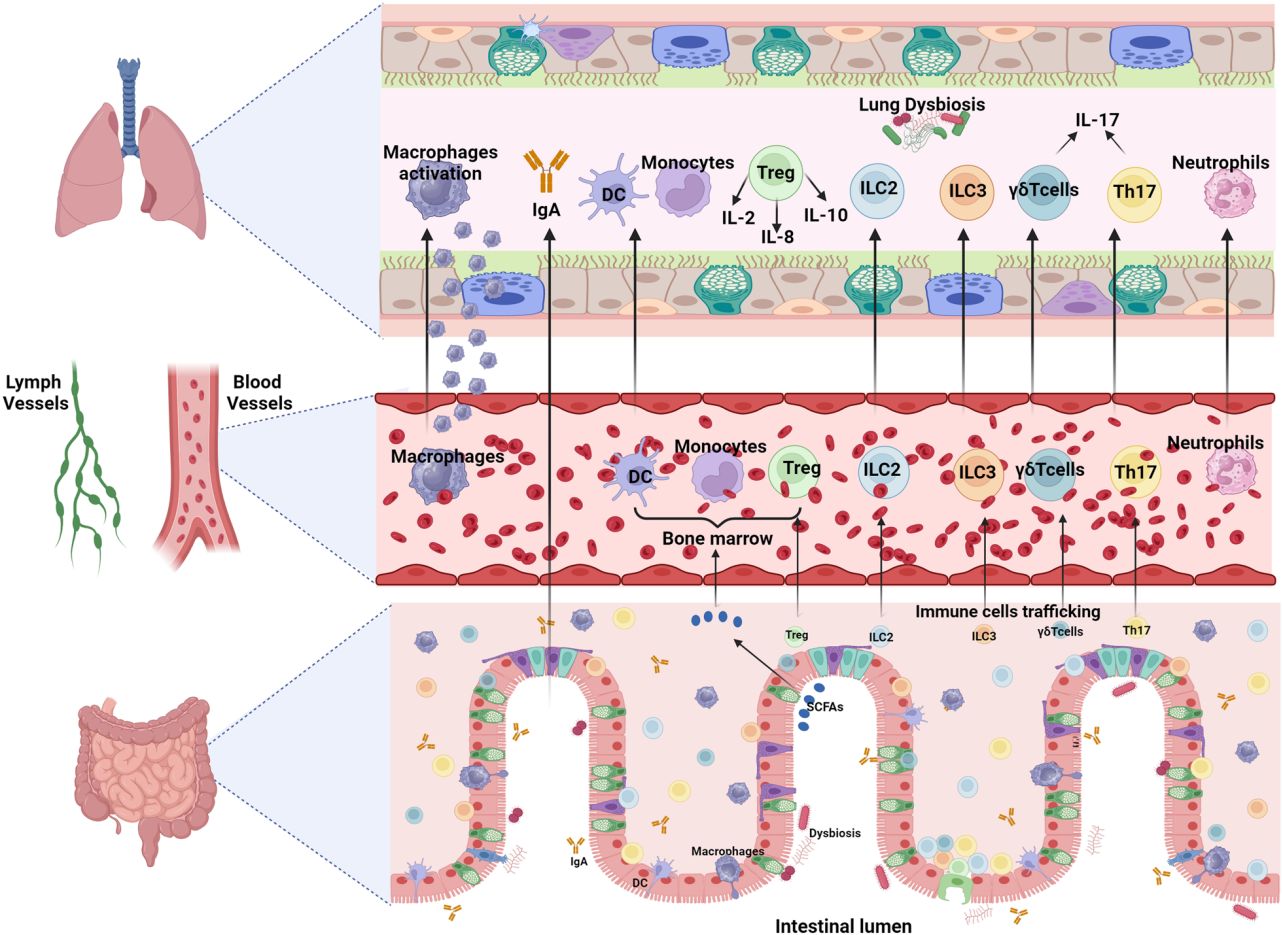
The migration of cells from the gut to the lungs has the potential to impact respiratory immunity, as illustrated by the movement of Tregs, Th17 cells, ILC2s, ILC3s, and γδ T cells from the gut to the lungs. Moreover, SCFAs derived from dietary fibers enter the peripheral circulation and bone marrow, playing a role in immune cell development, including Treg cells, which contribute to lung immune responses. Elevated Th17 levels in both alveolar and circulating compartments correlate with enhanced proportions of alveolar neutrophils, increased alveolar permeability, and organ dysfunction in ARDS. Finally, IgA derived from the gut might play a role in regulating immune responses beyond the mucosal tissues of the lung. *ARDS* acute respiratory distress syndrome, *IgA* immunoglobulin A, *ILC* innate lymphoid cells, *SCFAs* short-chain fatty acids, *Th17* T helper 17, *Treg* T regulatory cells

ALI manifests as hypoxaemia and enhanced alveolo-capillary permeability, leading to acute respiratory failure with a notable mortality rate. The immune system, particularly Tregs, plays a strategic role in the transition from injury to resolution in survivors of acute ALI/ARDS, with increasing evidence indicating that Tregs contribute to the suppression of lung inflammation [85]. Tregs have the capacity to mitigate inflammation-induced tissue damage through various indirect mechanisms, including their anti-inflammatory properties through suppression of macrophage anti‐inflammatory cytokine secretion [38, 39] and antiapoptotic capabilities [86], which contribute to establishing a conducive immune microenvironment for tissue repair and regeneration [87]. Treg cells exert anti-inflammatory effects through direct cell–cell interactions or by releasing cytokines like IL-10 or transforming growth factor (TGF)-β, with their differentiation dependent on the transcription factor FoxP3 [88, 89]. Moreover, acting as a “cytokine sink,” Tregs have the ability to capture specific effector cytokines [90]. Recent studies investigating the pathophysiology of ARDS have validated that the direct instillation of human umbilical cord mesenchymal stem cells into the lungs results in an increase of the alveolar Tregs, which was associated with the regulation of pro- and anti-inflammatory factors, including tumor necrosis factor (TNF)-α [91]. Moreover, it has been shown that the Tregs/CD4+ ratio in the bloodstream of ARDS patients is significantly higher compared to non-ARDS patients, while at the alveolar level, the Tregs/CD4+ ratio is reduced [92, 93]. Equivalent research has revealed a notable decline in CD4+, CD8+, and B lymphocyte counts, activation of Th1 and Th2 pathways, cytotoxicity, apoptosis, and endothelial dysfunction in ARDS patients. Interestingly, changes in the intestinal microbiome composition correlate with fluctuations in lymphocyte counts [93–95]. In addition, indirect evidence proposes that Tregs may influence IL-2 [96] and IL-8 [97] (Fig. 1). Moreover, Wang et al. (2012) investigated the chemotactic receptor for leukotriene B4 (LTB4) in the recruitment of Tregs to bronchoalveolar lavage fluid (BALF) in LPS-induced ALI. They examined BLT1 expression in both mouse and human Tregs and assessed its role in Treg migration both in vitro and in vivo. The research demonstrated that BLT1 plays a novel anti-inflammatory role in resolving ALI by mediating the alveolar recruitment of Tregs, suggesting that therapies disrupting the LTB4-BLT1 pathway after ALI onset could hinder recovery [98]. Additionally, an experimental study using a mouse model of LPS-induced ALI demonstrated that Tregs play a critical role during lung injury resolution by altering innate immune responses, indicating possible therapeutic targets for ALI treatment [99]. Indeed, Treg cells, crucial regulators of immune responses through cell-to-cell contact and secretion of inhibitory cytokines such as IL-10 and TGF-β1, infiltrate the lungs and may contribute to the pathogenesis of ARDS. They accumulate in the BALF of mice and patients with ALI, promoting the resolution of ALI by inducing TGF-β1 secretion and neutrophil apoptosis [99].

Further research using a murine model of lung injury has demonstrated that Tregs play a significant role in resolving ALI fibroproliferation by decreasing fibrocyte recruitment through the chemokine (C-X-C motif) ligand 12 (CXCL12)–CXCR4 axis [100].

On the other hand, clinical and experimental studies highlight the essential involvement of pro-inflammatory Th17 cells in initiating pulmonary inflammation and the cardinal role of the imbalance of Th17/Treg in the progression of the disease [101]. Th17 cells, a subset of CD4+ T lymphocytes, contribute to both the pathogenicity and immunoprotective mechanisms observed in diverse autoimmune diseases and play a crucial role in generating an inflammatory milieu during infections [102–104] by producing mainly cytokine IL-17 and, to a lesser degree, other inflammatory mediators like IL-6, TNF-α, and granulocyte–macrophage colony-stimulating factor [88]. Accumulating data demonstrate that Th17 represents a “double-edged sword” paradigm where Th17 has a fundamental function in eliminating pathogen eradication but concurrently contributes to lung damage via neutrophil recruitment [105]. In a nematode infection model, the central role of IL-17A in eliminating parasites was related to lung damage via neutrophil recruitment [106]. Moreover, in murine models of lung injury induced by Influenza A (H1N1) infection, the involvement of Th17 was associated with an early and appropriate immune response to pathogens while simultaneously fostering pathological inflammation in the lung [107, 108].

Previous research illustrated a substantial activation of Th17 cells during the initial phase of ARDS and an alleviation of the severity of ARDS by mitigating the Th17 response [109]. Moreover, it is well established that elevated levels of CD4+ T cell activation and proliferation, coupled with the presence of Th17 cells, mediate inflammatory cascades in the lungs, contributing to the pathophysiology of ARDS [110, 111]. Furthermore, enhanced levels of Th17 in alveolar and circulating compartments are associated with increased proportions of alveolar neutrophils, enhanced alveolar permeability, and organ dysfunction in ARDS [105, 112]. Moreover, a recent experimental study using a mouse model of LPS-induced ARDS has highlighted that IL-33 production increases the Th17/Treg ratio. At the same time, IL-33 deficiency eliminates the differentiation of T cells into Th17 cells, thereby restoring Th17/Treg balance. As a result, IL-33 deficiency notably suppresses inflammation, whereas treatment with recombinant IL-33 exacerbates lung inflammation [101]. These findings are in line with previous experimental research underscoring the diverse role of IL-33 in shaping the development and maintenance of the Th17 immune response by influencing proinflammatory Th17 cells in the small intestine to transition into a regulatory phenotype with immunosuppressive properties [113]. Interestingly, IL-33 has been demonstrated in experimental studies to directly suppress the expression of tight junction-related proteins both in vitro and in vivo, while also significantly increasing intestinal permeability in vivo, promoting bacterial translocation and exacerbating systemic and colonic inflammation [114, 115]. Finally, probiotic intervention demonstrates preventive effects against experimental particulate matter (PM)2.5-induced lung injury. Indeed, analysis of 16 S rRNA sequences revealed that probiotic treatment in a rat lung damage model induced by PM2.5 exposure could diminish microbiota abundance and diversity, enhance the presence of potentially beneficial bacteria, and diminish the levels of bacteria linked to inflammation. This effect was attributed to the suppression of inflammatory responses, modulation of Th17/Treg balance, and preservation of intestinal internal environment stability, indicating the pivotal contribution of intestinal Th17/Treg in the pathophysiology of ALI [116].

The generation, proliferation, and survival of Tregs are significantly influenced by host-derived nutrients and hormones [117]. Certainly, dietary components such as probiotics and prebiotics have the ability to impact health by altering the composition and function of the mucosal immune system and gut microbiota. Through competition or inhibition of adherence, they can increase pathogen exclusion and enhance intestinal epithelial integrity. Furthermore, both the gut and systemic immune systems can be influenced by these dietary factors, particularly by stimulating various components of the innate and adaptive immune responses. This involves the activation of regulatory T and B cells (Bregs), Th1, Th2, and Th17 responses, alongside the humoral response [118, 119]. Moreover, metabolites derived from commensal microbiota, including SCFAs, play a crucial role in regulating Treg homeostasis and function within the gut-associated lymphoid tissue (GALT) (Fig. 1) [117]. SCFAs stand out as potentially significant. Originating from the processing of dietary fiber by gut microbiota, SCFAs emerge as the primary intestinal metabolic elements by regulating immune reactions, including the differentiation, activation, and recruitment of immune cells (Fig. 1). Additionally, SCFAs demonstrate immunomodulatory properties that extend beyond exerting effects solely within the gut. Recent research findings suggest SCFAs as potential therapeutic strategies to reduce Th17 cell differentiation while simultaneously boosting Treg cells and enhancing their suppressive potential [120]. Indeed, SCFAs trigger the release of TGF-β1 by hindering histone deacetylase-mediated activating protein-1 activation within intestinal epithelial cells (IECs). Consequently, this process promotes the generation of Tregs and elicits anti-inflammatory, immune effects [121–123].

Given the restricted therapeutic strategies in the management of patients with ALI/ARDS, enhancing our understanding of the functions of Tregs and Th17 in the pathophysiology of ALI/ARDS holds the potential to prepare the way for the development of innovative treatments for affected individuals.

### γδ T cells

Γδ T cells represent a distinctive subpopulation of Th17 lymphocytes located at epithelial surfaces, including the intestine and the lung, playing a strategic role in the modulation of immune responses against microbial pathogens [124, 125]. Despite γδ T cells being most frequent in the lamina propria/smooth muscle of the airways and less so in the alveolar epithelium, their localization in the subepithelium of alveolar regions within the lung [126] implies a role in influencing the immune response against bacterial infections [127], among others, through their ability to regulate macrophage homeostasis and activation [128]. This is supported by experimental studies showing that in individuals deficient in γδ T cells, total lung inflammation and alveolar-capillary leak were enhanced and that γδ T cells protected against LPS-induced lung inflammation and alveolar-capillary dysfunction [99, 129, 130].

Furthermore, it is highlighted that modifications in mucosal barrier function associated with enhanced permeability have been linked to bacterial translocation and immune dysfunction. This alteration has a causal connection with several pathological conditions, including inflammatory bowel disease and ischaemic stroke. Additionally, increased intestinal permeability triggers intestinal inflammatory responses and may, in part, contribute to the transition from protective γδ T cells to pathogenic γδ T cells [83, 131–134]. Very recently, an experimental study in a murine model of stroke demonstrated that γδ T cells, rather than CD4^+^ Th17 cells, serve as a principal source of IL-17A in the injured brain and lung following stroke. Importantly, it is noteworthy that the migration of γδ T cells from the small intestine to the brain and lung plays a pivotal role in exacerbating stroke and lung injury. Additionally, the subdiaphragmatic vagus nerve actively participates in mediating the migration of small intestine-derived γδ T cells into the brain and lung after a stroke [131]. Moreover, a current experimental study of lung infection with *Ps. aeruginosa* found that prophylactic nutritional intervention with inulin supplementation triggers a higher proportion of γδ T cells in the blood, accompanied by a higher infiltration of IL-17-producing γδ T cells within the lungs [135]. Finally, the crucial role of γδ T cells in the gut-lung crosstalk is indicated by studies underscoring the elevated susceptibility to chronic inflammatory lung disease observed in individuals with inflammatory bowel disease [136]. Exploring the foundational role of γδ T cells in the communication between the gut and the lung would provide insights into whether adjusting the migratory behavior of γδ T cells could offer an additional therapeutic avenue to mitigate the onset or advancement of ALI resulting from the disruption of intestinal barrier function.

### Antibody-secreting cells

The regulation of humoral immunity involves a special population of B cells with the capacity to produce and release substantial quantities of antibodies, known as antibody-secreting cells (ASC) [137]. Within the gastrointestinal milieu, a combination of foreign diet antigens, commensal microbiota, and intermittent harmful pathogens results in the consistent differentiation of B cells into ASC. This sustained immune response establishes the gut as the residence for over 80% of mammalian ASC predominantly expressing IgA [138, 139]. Playing a central role in local immunity, IgA serves as the primary antibody within the mucosal immune system. It is essential for resisting the invasion of external pathogenic microorganisms, neutralizing bacterial toxins, and contributing to the establishment of the immune barrier and immune clearance while mediating immune responses, which can influence the progression of autoimmune diseases [140, 141]. Current research indicates that ASC derived from the gut plays a role in regulating immune responses beyond mucosal tissues, including in the blood, central nervous system (CNS), and the kidney [139]. To our knowledge, the data regarding the role of IgA in the pathophysiology of ALI/ARDS are scarce. This scarcity can be attributed to the fact that secretory IgA, primarily produced by mucosa-associated lymphoid tissue, is the main immunoglobulin in the upper airways. In contrast, IgG is the principal immunoglobulin in the alveolar spaces, mainly entering through passive diffusion from the systemic circulation. At the bronchial surfaces, IgM, also derived from mucosa-associated lymphoid tissue, is dominant and enhances pathogen opsonization by activating the complement system [142, 143]. However, research investigating the pathophysiology of other inflammatory lung diseases characterized by chronic neutrophil infiltration, such as COPD, indicates an impaired lung IgA immune function and a correlation between the decrease in IgA levels and the severity of the disease [144–146]. Moreover, recent data in patients with severe acute respiratory syndrome coronavirus 2 (SARS-CoV-2) infection proposes a dual nature of IgA’s impact on the disease progression. In the early stages, IgA-induced Neutrophil Extracellular Traps (NET) release is posited to be beneficial, acting as a defense mechanism against SARS-CoV-2 entry into the mucosal region. However, as the disease advances, NET release may become detrimental, potentially exacerbating tissue damage [140].

### Innate lymphoid cells

The innate lymphoid cells (ILCs) are categorized into three main groups: ILC1, ILC2, and ILC3, each comprising multiple sub-populations [147]. Recent evidence highlights that the intestinal microbiota may influence lung immune responses in newborn mice, particularly in combating pneumonia, by triggering the production of IL-22 derived from ILC3 in the lungs of animals (Fig. 1) [148]. In addition, ILCs are found to play crucial roles in lung injury, lung allergies, fibrosis, and pulmonary infections [149–152]. Accumulating evidence suggests that ILC2s present in the lung may derive from the intestine through circulation (Fig. 1). Huang and co-workers (2018) reported that ILC2s, originating from a resting state in the lamina propria of the intestine, enter the lymphatics and recruited into the lung through circulation as a result of sphingosine 1-phosphate (S1P)-mediated chemotaxis [153]. Moreover, using integrated microbiota dysbiosis approaches, Pu and co-authors reveal that the gut microbiota plays a role in guiding the migration of ILC2s from the gut to the lung, establishing a gut-lung axis. Specifically, they identified *Proteobacteria* as the pivotal species within the gut microbiome that supports the natural migration of ILC2s, with elevated *Proteobacteria* levels leading to increased IL-33 production [154]. Nevertheless, as the pathophysiology of migration of ILC2s within the lung during inflammatory conditions remains mainly unclear and there is limited knowledge about the signals that could potentially regulate this process, further studies are needed to investigate the plausible participation of ILCs of intestinal origin in the pathogenesis of ALI/ARDS.

## Areas of further research

### Fecal microbiota transplantation

Fecal microbiota transplantation (FMT) aims to restore the functions of an altered gut microbiota by introducing fecal material from a healthy donor. Interest in this method, which has well-established benefits for treating conditions such as food poisoning and dysentery over centuries, surged in 2013 after the publication of a randomized control trial that demonstrated FMT's substantial superiority over standard care for treating recurrent *Cl. difficile* infections (CDI) [155, 156]. Notably, in critically ill patients with severe and complicated CDI, rescue FMT emerged as a promising alternative to surgical treatment, achieving a primary cure rate of nearly 80%, thereby enabling almost 90% of patients to avoid colectomy [157]. However, there is limited evidence regarding the use of FMT in managing critically ill patients with antibiotic-associated diarrhea (AAD) caused by pathogens other than *Cl. difficile* or those of unknown origin, which account for about two-thirds of AAD cases [158–161]. Yet, in a case series studying the use of FMT in critically ill patients with AAD, Dai, and coworkers (2019) described good clinical outcomes without infectious complications [161]. Moreover, experimental research in recent years has suggested that by maintaining the balance of pulmonary flora and altering the structure and diversity of both pulmonary and intestinal microbiota, FMT might effectively prevent and treat chronic respiratory diseases through the regulation of the pulmonary and the intestinal flora [162]. Furthermore, the effectiveness of FMT, as highlighted by clinical and experimental studies in CDI, might arise from a combination of direct microbiological actions against *Cl. difficile* and indirect mechanisms, such as the production of microbiota-derived metabolites like secondary bile acids and SCFAs. FMT appears to modulate the intense inflammatory response triggered by *Cl. difficile* by involving Tregs, which play a crucial role in reducing various cells and soluble inflammatory mediators [163], making it a significant treatment modality for ARDS patients. However, considering potential complications such as sepsis and exacerbation of inflammatory processes, further research is necessary to ensure safety and efficacy in critically ill ARDS patients [164].

The aforementioned findings hold promise for the development of successful therapies to manage critically ill patients, including those with ARDS. However, caution is warranted, as not all experimental results from animal models translate directly to humans. Further research is needed to explore the relevance of these findings in the management of critically ill patients.

## Conclusions

In summary, the pleiotropic relationship between the gut microbiota and immune cells plays a crucial role in maintaining immune balance by impacting systemic responses, including those seen in ALI/ARDS. Treg cells offer protection by suppressing lung inflammation, while Th17 cells can worsen lung damage. SCFAs from the gut microbiota, probiotics, and FMT emerge as potential therapies, influencing immune cell function, gut dysbiosis, and intestinal barrier integrity. Additionally, γδ T cells, ASCs, and ILCs play roles in lung immune responses, with implications for ALI/ARDS. Understanding these interactions offers hope for innovative treatments to address the urgent need for effective ALI/ARDS therapies.

## Data Availability

Not applicable.
